# Activity Performance, Participation, and Quality of Life Among Adults in the Chronic Stage After Acquired Brain Injury—The Feasibility of an Occupation-Based Telerehabilitation Intervention

**DOI:** 10.3389/fneur.2019.01247

**Published:** 2019-12-06

**Authors:** Aviva Beit Yosef, Jeremy M. Jacobs, Shira Shenkar, Jeffrey Shames, Isabella Schwartz, Yehudit Doryon, Yuval Naveh, Fatena Khalailh, Shani Berrous, Yafit Gilboa

**Affiliations:** ^1^School of Occupational Therapy, Faculty of Medicine, The Hebrew University of Jerusalem, Jerusalem, Israel; ^2^Faculty of Medicine, Department of Geriatrics and Geriatric Rehabilitation, Hadassah Medical Center, Hebrew University of Jerusalem, Jerusalem, Israel; ^3^Occupational Therapy Department, Maccabi Health Services, Jerusalem, Israel; ^4^Medical and Health Professions Division, Maccabi Health Services, Tel Aviv-Yafo, Israel; ^5^Faculty of Medicine, Physical Medicine and Rehabilitation Department, Hadassah Medical Center, Hebrew University of Jerusalem, Jerusalem, Israel; ^6^Occupational Therapy Department, Medical and Health Professions Division, Maccabi Health Services, Tel Aviv-Yafo, Israel; ^7^Maccabi Health Care Services Group, Occupational Therapy Department, Bayit Balev Hospital, Bat Yam, Israel; ^8^Occupational Therapy Department, Hadassah Medical Center, Jerusalem, Israel

**Keywords:** chronic acquired brain injury, activity performance, participation, neurorehabilitation, telerehabilitation, cognitive orientation to daily occupational performance approach, metacognitive approach, occupational therapy

## Abstract

**Objective:** Acquired brain injury (ABI) is a leading cause of long-term disability. This calls for effective and accessible interventions to support participation in the community over time. One promising avenue to answer this need is telerehabilitation. Prior to conducting a larger trial, the main objective of this pilot study is to explore the feasibility, acceptability, and preliminary efficacy of a metacognitive occupation-based intervention in a telerehabilitation format with adults and older adults in the chronic phase after ABI.

**Methods:** Five community dwelling participants (ages 65–72), 6–10 months post-ABI, with scores 2–4 on the modified Rankin scale and without dementia, completed the teleintervention. The intervention included ~10 weekly videoconferencing sessions administered by an occupational therapist using the Cognitive Orientation to Daily Occupational Performance approach. Each participant defined five functional goals and three were trained and two were not trained during the intervention. Evaluations were conducted at pre, post, and 3-month follow-up. The primary outcome measures included activity performance (The Canadian Occupational Performance Measure; COPM), participation (the Mayo-Portland Adaptability Inventory-4 Participation Index; MPAI-4-P), and quality of life (QoL) (stroke impact scale; SIS). Other measures included a feedback interview, satisfaction questionnaire, field notes, and a treatment fidelity checklist.

**Results:** The teleintervention was found to be feasible and the participants expressed a high degree of satisfaction with the intervention and the technology use. A Wilcoxon Signed-Ranks test indicated statistically significant improvements post intervention in COPM performance (*z* = −2.023, *p* = 0.043) and satisfaction (*z* = −2.023, *p* = 0.043) ratings. Additionally, clinically significant improvements (≥2 points) in both performance and satisfaction with performance were found for each participant in at least three of their five defined functional goals. Trends toward significant improvement were found in MPAI-4-P ratings post intervention (*z* = −1.826, *p* = 0.068). Furthermore, clinically significant improvements (≥15 points) post intervention were found for each participant in some subscales of the SIS. Results were partially maintained at 3-month follow-up.

**Conclusions:** This pilot study demonstrated the feasibility of a metacognitive occupation-based telerehabilitation intervention and its potential benefits in activity performance, participation, and QoL for older adults coping with long-term disability following ABI.

**Clinical Trial Registration**: www.ClinicalTrials.gov, identifier: NCT03048708.

## Introduction

Acquired brain injury (ABI) is a major health issue and a leading cause of disability worldwide (1, 2). ABI is defined as brain injury that occurs after birth, and the most common types of ABI are traumatic brain injury (TBI) and stroke (3). ABI can cause a variety of impairments depending on the affected brain area and the severity of the damage. It may manifest in sensory, motor, cognitive, behavioral, and emotional impairments (4–6) and lead to long-term functional limitations and participation restrictions in daily life (3, 7–11).

There is evidence that home and community-based rehabilitation after ABI is effective in reducing disability (12–14), and that longer-term rehabilitation programs lead to improved global outcomes such as social participation and quality of life (QoL) (15). In addition, there is evidence supporting the use of occupation-based interventions to improve daily activity performance (16). However, most studies currently continue to emphasize measurement and intervention in terms of motor function and mobility. Therefore, further research focusing on achieving meaningful participation and improving QoL, extending beyond impairment remediation approaches, is still needed (10, 17).

Furthermore, most rehabilitation resources are invested in the first weeks and months following the ABI, at the sub-acute stage, while less emphasis is placed on long-term interventions (3, 6, 18). There is a lack of sufficient rehabilitation services in the community offering continued support in the chronic stage (6, 12, 19–21). This might be explained by low accessibility and availability as well as numerous barriers to community-based rehabilitation services, while high costs and reimbursement issues limit the possibility of receiving in-home rehabilitation services (20, 22–24). Many ABI survivors therefore continue to live in the community with limited participation in meaningful daily activities (8, 25–27). Accordingly, there is a growing understanding that ABI is a chronic health condition that requires long-term attention, and necessitates the improved continuity of both short and long-term rehabilitation services in the months and years following brain injury (11, 18, 28–32).

This need for developing cost-effective and accessible intervention models for people in the chronic phase following ABI in order to facilitate participation and community reintegration (6, 29) can be addressed by telerehabilitation. Telerehabilitation is defined as the use of information and communication technologies to provide remote rehabilitation services to people in their homes or other environments (33). Telerehabilitation has a wide range of delivery modes from texting or talking on mobile phones to video conferencing and to more complex systems like virtual reality video games. One strong advantage of telerehabilitation is that it allows services to be delivered to people in their homes without the therapist being physically present with them. This can clearly improve accessibility and cost effectiveness. However, there may also be challenges such as difficulties using the technologies, privacy issues, and the attitudes of patients and clinicians (23, 34–36).

Supporting evidence can be found in the literature for telerehabilitation interventions with adults after ABI in terms of feasibility and patient satisfaction (37–40). A recently published systematic review that evaluated the efficacy of telerehabilitation interventions among stroke survivors concluded that it may have equal or even better outcomes than face-to-face interventions in motor function, cognitive function, and emotional state (41). However, other systematic reviews and meta-analyses concluded that the evidence available is inconclusive and insufficient (22, 42–44). It should be noted that most studies that examined the efficacy of telerehabilitation programs included interventions that focused on body functions on the impairment level such as balance, upper-extremity function, and cognitive and language skills (37, 38, 41, 45–51).

To the best of our knowledge, there have not been many studies that have examined telerehabilitation interventions for individuals in the chronic phase after ABI that focused directly on the activity and participation level. Chumbler et al. (52) evaluated a telerehabilitation program that focused on improving functional mobility among 52 veterans post-stroke. Results indicated that the intervention significantly improved physical function. However, the focus of the intervention was limited to functional mobility, and it did not address broader domains of daily function. Another trial (53) evaluated the efficacy of a telephone-delivered problem-solving treatment. The results suggested this intervention offers the promise of reducing psychological distress after combat-related mild TBI. The sample in this study included 356 young soldiers (mean age 29.35) who had been exposed to stressful combat situations, thus not allowing the generalization of the results to a broader ABI population. A third randomized controlled study (*N* = 38) evaluated the effects of an errorless learning training approach in comparison with a didactic strategy instruction approach, both delivered over the telephone, on the reported everyday memory problems of adults with chronic TBI. Results demonstrated that both of these treatment approaches improved reported everyday memory functioning (54). This study was limited to memory-related goals with a focus on a specific technique of errorless learning. Other studies included a feasibility study (55), case-study (56), and pilot studies (24, 57, 58).

Ng et al. (24) used the Cognitive Orientation to Daily Occupational Performance (CO-OP) approach via video sessions in their study with three adults after TBI (ages 34–55). The results demonstrated a high level of satisfaction among the participants, an improvement in their level of performance and satisfaction in functional goals, and a trend toward greater community participation. Despite the preliminary nature of this evidence (24), it sparked our interest due to the occupation-based, client-centered intervention approach that was used. The CO-OP approach is a metacognitive approach that focuses on strategy training and problem solving to improve the performance of daily activities, as opposed to training directed at improving the underlying impaired body functions. Essential elements of this approach are client-chosen functional goals, dynamic performance analysis, use of global and domain-specific strategies, and a process of guided discovery with enabling principles. The approach uses a global problem-solving strategy, “Goal-Plan-Do-Check,” that outlines four steps toward achieving goals: setting a specific functional goal, creating a plan that includes steps to achieve the personal goal, executing that plan, and checking if the plan was executed and if it worked (59). The CO-OP was adapted for use in different populations including adults with ABI (60). Several studies have demonstrated the efficacy of using the CO-OP approach to promote functional goals for individuals during the chronic phase after ABI (60–65).

To summarize, there is a need for an improvement in the continuum of rehabilitation services provided to ABI survivors that will enable meaningful participation and community reintegration (6, 10, 17, 29). The CO-OP approach is an appropriate treatment option to meet this need, consistent with existing recommendations for practice guidelines in the chronic phase after ABI (12, 16, 30). The use of the CO-OP approach through remote rehabilitation enables the application of the intervention in an accessible manner in the home environment, with potential for long-term, cost-effective treatment (66). Although this has been shown in a small pilot study to be both feasible and potentially effective (24), it should be noted that the evidence is preliminary and limited to younger adults with a diagnosis of TBI. It is likely that a more comprehensive and definitive understanding of this innovative treatment modality would be gained by undertaking a sufficiently powered randomized controlled study. Therefore, prior to conducting a larger trial, the main objective of this pilot study is to explore the feasibility, acceptability, and preliminary efficacy of the CO-OP approach in a telerehabilitation format with adults and older adults in the chronic phase after various types of ABI. We had three specific research questions: (1) Is the intervention feasible in terms of recruitment, retention and intervention adherence, fidelity of treatment, and technology delivery? (2) Will the intervention be acceptable to the participants and their significant others? and (3) What is the effect of the intervention program in improving activity performance, participation, and QoL?

## Materials and Methods

### Design

This study was a quasi-experimental pilot study. The study protocol was approved by the research ethics committees of Hadassah-Hebrew University Medical Center, Jerusalem, and Maccabi Healthcare Services, Bat-Yam, Israel (ethical committee registration numbers: 0689-15-HMO and 192016, respectively).

### Participants

Community-dwelling adults with ABI were recruited between February 2017 and April 2018 from a day-rehabilitation hospital unit and from 2 day-rehabilitation clinics in and around Jerusalem and Bat-Yam, Israel. There were several inclusion criteria: (1) at least 6 months post-ABI, reflecting the chronic rehabilitation phase, (2) aged 18 years and over, (3) sufficient proficiency in Hebrew or English to undertake the study, (4) slight to moderately severe disability in daily function, based on the modified Rankin scale (mRS) scores of 2–4 (67, 68), (5) ability to identify at least 3 day-to-day functional difficulties that they experienced on which to base treatment goals, (6) internet access in their home, and (7) having a significant other who knows the participant well, is at least 18 years old, and who expressed a willingness to be involved in the study. The presence of the significant other in the sessions was not an eligibility criterion. There were some exclusion criteria: (1) dementia diagnosis or Mini Mental Status Examination (MMSE) < 24 (69) or Montreal Cognitive assessment (MoCa) < 19 (70), (2) moderate or severe aphasia, and (3) an acute or chronic illness that has a significant impact on the ability to cooperate in the study.

### Procedure

After obtaining the approval of the research ethics committees, potentially eligible participants were identified by occupational therapists (OTs) who worked in the rehabilitation departments. Patients who were interested in participating were referred to the research coordinator (Author ABY) who contacted them and further screened for eligibility. The participants and significant others were informed about the study process as well as the technology requirements. Eligible patients who agreed to participate gave written informed consent in accordance with the latest declaration of Helsinki. The study period lasted ~6 months for each participant and started after the completion of the occupational therapy treatment in the day-rehabilitation clinics. The study procedure included different steps: (1) baseline assessment, (2) 3-month intervention period, (3) post intervention assessment, and (4) 3-month follow-up assessment.

The baseline assessment was performed in two sessions (~2 h overall). These sessions were conducted by the same OT that provided the intervention and were done face-to-face in the participant's home in order to establish a therapeutic relationship as the basis for the remote intervention sessions that followed. In addition, the baseline assessment meeting ended with training in the use of the technological equipment. The post intervention assessment and 3-month follow-up assessment were also conducted face-to-face at the participants' homes, with the exception of participant 1 who did these assessments via a phone call. Assessments and intervention sessions were performed by licensed OTs (authors ABY and SS) with more than 5 years of experience in geriatric and neurological rehabilitation. Both OTs are certified in the CO-OP approach after attending the standard CO-OP workshop, and they trained together in the administration of the measures. For each participant, the OT who carried out the baseline assessment was the same one who performed the intervention program. The post intervention and follow-up assessments were conducted by the other OT in order to prevent bias.

### Intervention

The intervention program included up to 15 remotely delivered CO-OP sessions, 1–2 times a week (~45 min per session), and they were spread out over a 3-month period. The intervention was administered in a telerehabilitation format via video conferencing using Skype™ software. Skype™ is free, available and easy to use, and security for the users is insured by encryption of this software program (71). The video sessions occurred while the participant was at home, in a location they preferred, and the OT was alone in her office to ensure privacy. At the beginning of the process the OT explained that the significant other's involvement was important for two aspects of the process. The first aspect was supporting the therapeutic process in line with the CO-OP principles (59). This was especially significant in both facilitating the execution of plans during the week between the sessions and the generalization and transfer of strategies and skills to the participants' daily routines. In some cases, the significant other was part of a plan the participant formulated for achieving a goal (e.g., my wife will drive me to the community center). The second aspect was supporting the logistics of the intervention. Since the therapist is not physically present in the room, the presence of another person is necessary for safety reasons in cases of actual performance of specific activities during the sessions (e.g., cutting vegetables in the kitchen). In addition, in some cases, the significant other assisted with the use of the telerehabilitation technology. The video sessions were recorded using TalkHelper Call Recorder for Skype software and were stored in a local secured hard drive. In addition, after each session, the OT documented key points in field notes. Participants who did not have a computer or tablet at home were provided with iPads.

The first phase of CO-OP intervention is defining client-chosen functional goals (59). In this study, each participant identified five functional goals during the baseline assessment using the Canadian Occupational Performance Measure (COPM), of which three were the focus of the intervention (trained goals). The other two goals were not addressed directly during the intervention sessions (untrained goals), to allow assessment of generalization and transfer of learning. At the first intervention session, the OT and participant reviewed and re-discussed the goals, and the OT taught the participant the global problem-solving strategy (Goal-Plan-Do-Check). In the proceeding sessions the OT guided the participant in the use of this strategy to help them discover their performance problems as well as potential task-specific strategies to improve their performance and enable goal attainment. Rather than providing the participant with the solutions, the OT facilitated this process with questions and feedback. The CO-OP is a performance-based approach. Therefore, the participants actually performed some of the activities during sessions if it was possible, and the OT observed that actual performance in the participant's natural environment via video conferencing. In some cases, it wasn't possible to perform the activity online, either because it was done in other settings (e.g., a community center), due to privacy issues (e.g., dressing), or for safety reasons (e.g., peeling vegetables while the significant other was not present). In these cases, the sessions included discussing the performance, the plans, and strategies. Each participant received a folder with materials to support the intervention. To ensure adherence to the CO-OP protocol, meetings were held regularly between ABY, SS, and YG to review and discuss the intervention sessions.

### Outcome Measures

#### Socio-Demographic and Clinical Characteristics

Participants' socio-demographic characteristics were documented and included information such as age, years of education, and the identity of the significant other. Clinical information included the type and side of the ABI, time since the ABI, cognitive screening test scores, and functional status. The information was collected through a review of the medical records and through a conversation with the participants at the baseline assessment. Reports regarding other outpatient treatments were documented at post intervention and follow-up assessments.

#### Feasibility

Therapists' field notes and recordings from the intervention sessions were used to assess feasibility aspects. Information regarding eligibility, recruitment, and retention rates was documented. In addition, intervention adherence was described by the number of participants who completed the intervention program (with a minimum of eight sessions), the number of sessions completed, and the duration of each intervention session. To assess fidelity of treatment, video-recordings and field notes from three sessions for each participant were reviewed and scored using the CO-OP fidelity checklist (72, 73). In addition, the quality of the online communication as well as specific technical problems that arose (e.g., video and audio disruptions, the need of assistance to operate the software and equipment, and problems with the internet connection) were also documented.

#### Acceptability

Acceptability of the intervention was assessed at post intervention by a satisfaction questionnaire completed by the participants and a short semi-structured feedback interview with the participants and their significant others. The questionnaire was developed for this study and included 13 statements (detailed in Supplementary Figure 1) that were rated on a 5-point scale (from 1, being very low, to 5, being very high). The questionnaire assessed satisfaction with the intervention in general and other aspects such as the remote delivery, the technology use, and the therapeutic relationship. The short semi-structured feedback interview included two main questions: (1) What are the main benefits you experienced while participating in the program? and (2) What are the main challenges you experienced while participating in the program? Follow-up questions were added to encourage elaboration on these topics. The interviews were audio-recorded and later transcribed.

#### Preliminary Efficacy

##### Primary outcome —activity performance in participant-chosen goals

Activity performance was measured with the Hebrew version of the Canadian Occupational Performance Measure (COPM) (74). The COPM measures the client's perceptions of their performance of daily activities over time and facilitates client-centered goal setting as the basis of the intervention process. It is a semi-structured interview that helps the client identify occupational performance problems and then prioritize them using an importance rating scale (1: not important at all, 10: extremely important). The client then rates the five most important goals on 10-point rating scales of performance and satisfaction (1: not able to do it/not satisfied at all, 10: able to do it extremely well/extremely satisfied). Changes in the client's perception of their performance and satisfaction of two points or more is considered a clinically significant change. The COPM has demonstrated good validity, test–retest reliability, and sensitivity to change in many studies, and it is widely used as an outcome measure with various populations including adults after ABI (74–78). In this study, the COPM was administered as part of the baseline assessment and served as the basis for setting five goals. Three of these goals were directly addressed in the intervention process, and the other two goals were not.

##### Secondary outcomes—participation and quality of life

Participation was measured with the Mayo-Portland Adaptability Inventory-Participation index (MPAI-4-P) (79), using the Hebrew version (80). The MPAI-4 is a questionnaire widely used among rehabilitation professionals to evaluate the recovery progress among people after ABI (81). The MPAI-4 includes 29 items divided into three indexes: (a) Ability (e.g., motor, sensory and cognitive abilities), (b) Adaptation (e.g., emotional state and social interactions), and (c) Participation (e.g., leisure activities, work, and use of transportation). In this study, we used the participation index (includes eight items), which was completed by the participants. The items are ranked on a scale of 0–4, with a higher score indicating more participation difficulties and limitations. Item scores are calculated and converted to a standardized T-score that represents different levels of participation limitation: scores below 30 denote relatively good participation; 30–40 denote mild participation limitations; 40–50 denote mild to moderate participation limitations; 50–60 denote moderate to severe participation limitations; and scores above 60 denote severe participation limitations. The MPAI-4 has well-documented psychometric properties. Previous studies have described good internal consistency, construct and concurrent validity, as well as predictive validity (15, 81–83). In addition, the MPAI-4 was found to be sensitive to clinical change following rehabilitation (84, 85).

Quality of life was measured with the Stroke Impact Scale (SIS) (86) using the Hebrew version (87). The SIS is a questionnaire for evaluating the self-perceived effect of stroke on a wide range of domains and is commonly used as a measure of QoL. The questionnaire includes 59 items divided into eight subscales: limb strength on the affected side, memory and thinking, mood, communication, daily activities, mobility, hand function, and participation. The patient rates each item on a scale of 1–5, with a low score indicating more difficulty or limitations. In addition, there is another subscale with one item measuring general recovery on a scale of 0 (no recovery) to 100 (full recovery). The SIS does not have a total score, but rather a score for each subscale. Raw scores are converted to standard scores (between 0 to 100). A change of 15 points or more is considered a clinically significant change (88, 89). The SIS is widely used in research and is reliable, valid, and sensitive to changes (86, 88, 90–92). It had been used previously with individuals after stroke and TBI (93).

### Statistical Analysis

Statistical analyses were conducted using SPSS version 24.0 (IBM Corp., Chicago, IL, USA). Due to the small sample size, the results are presented for each participant separately as well as for the group, and both statistically and clinically significant changes are noted. Descriptive statistics were used to describe the participants' characteristics and the feasibility aspects of the study. Acceptability of treatment was analyzed with a combination of descriptive statistics and qualitative analysis of the feedback interviews with the participants and their significant others.

To analyze the preliminary efficacy of the intervention, we calculated the median and interquartile range (IQR) scores of the outcome measures at baseline, post intervention, and 3-month follow-up. Since the sample was small, non-parametric Wilcoxon signed-rank tests were used to detect statistically significant changes from baseline to post intervention and from baseline to follow-up with the *p*-value set at <0.05. In addition, an effect size (*r*) was calculated from the *z*-value of Wilcoxon signed-rank test ($r=z/\sqrt{}n$) (94) and can be interpreted as a small (*r* ≤ 0.10), medium (*r* = 0.30), and large (*r* ≥ 0.50) effect size (95).We did not make adjustments for multiple testing because in a pilot study there is more of a concern for a type II error than a type I error (65, 96).

## Results

Socio-demographic and clinical characteristics of the participants are presented in Table 1. The sample included older adults (age range 65–72 years), 6–10 months post-ABI, with stroke being the most common type of injury (80%). MRS scores ranged between 2 (slight disability) and 4 (moderately severe disability). The participants had 2–15 years of education, and they did not have dementia. Three participants had prior experience using a computer and/or tablet, and the other two participants did not. Three of the participants were first-time Skype™ users. We supplied an iPad for the intervention period for two participants who did not own a computer or a tablet. None of the participants received additional occupational therapy during the intervention period.

**Table 1:** Socio-demographic and clinical characteristics of the participants (*N* = 5).

**Characteristics**	***N* (%)**	**Mdn (IQR)**
Age		67 (65.5–69.5)
Sex		
Female	3 (60%)	
Male	2 (40%)	
Education (years)		11 (5.0–14.5)
Significant other		
Spouse	3 (60%)	
Child	2 (40%)	
Living situation		
Alone	2 (40%)	
With spouse	3 (60%)	
Previous use experience		
Computer/tablet	3 (60%)	
Skype^TM^	2 (40%)	
Type of ABI		
Hemorrhagic stroke	2 (40%)	
Ischemic stroke	2 (40%)	
Subdural hematoma	1 (20%)	
Side of ABI		
Right	1 (20%)	
Left	4 (80%)	
Time since ABI (months)		8 (6.5–9.0)
Cognitive status		
MMSE (*n* = 4)		26 (25.0–28.5)/30
MoCA (*n* = 1)		24/30
mRS scores		
Score 2	2 (40%)	
Score 3	1 (20%)	
Score 4	2 (40%)	

*Mdn, Median; IQR, interquartile range; ABI, acquired brain injury; MMSE, Mini Mental State Examination, cutoff score in this study was 24 (69); MoCA, Montreal Cognitive Assessment, cutoff score in this study was 19 (70); mRS, the modified Rankin Scale (97, 98), the scale runs from 0 to 6, running from perfect health without symptoms to death—0: no symptoms; 1: no significant disability; 2: slight disability; 3: moderate disability; 4: moderately severe disability; 5: severe disability; 6: dead. In this study we included participants with scores of 2–4*.

### Feasibility

#### Recruitment, Retention, and Intervention Adherence

**Figure 1:**
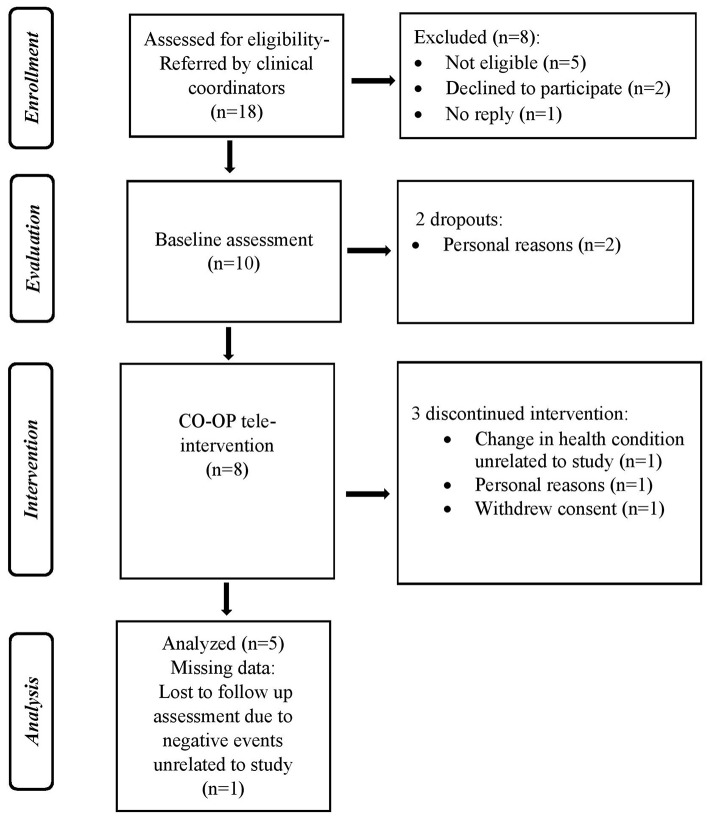
Flow diagram of the study enrollment, evaluation, intervention, follow-up, and analysis.

The flow diagram (see Figure 1) provides details regarding the process of enrolment, intervention, and assessments. During the recruitment period, 18 potentially eligible ABI patients were referred to the research director by clinical coordinators in the day-rehabilitation clinics and assessed for eligibility. Eight patients were excluded from the study; two declined to participate, five were found not eligible and one did not reply to the researcher's contact efforts (via phone calls and text messages). Among those referred, 55% (10/18) were eligible, agreed to participate, and started the study procedure. Two of the 10 consenting participants dropped out during baseline assessments. Five of the eight remaining participants completed the 3-month tele-intervention program and the post intervention assessments (62% retention rate). Those who discontinued the intervention withdrew from the study after one, three, and six sessions. The reasons for discontinuing the intervention are detailed in Figure 1. Participants who completed the CO-OP program received 8–14 intervention sessions (mean 10.6 ± 2.2 sessions). Four participants received the sessions over 3 months, while the fifth participant's intervention (participant 4) was extended to 8 months due to several hospitalizations unrelated to the study that led to breaks in the intervention program. Despite these breaks, the participant expressed high motivation to continue the program and good recovery after the hospitalizations. Therefore, he continued to participate in the intervention process. The average session length was 46.3 ± 12.4 minutes. Of the five participants who completed the intervention period, four participants completed the 3-month follow-up assessment. No adverse events related to the intervention were reported.

#### Fidelity of Treatment

In order to evaluate the treatment fidelity to the CO-OP approach, the CO-OP Fidelity Checklist was used (72, 73). This questionnaire was completed based on observing video recordings (that due to technical and ethical issues were available only for participants 3, 4, and 5) and reading the therapist's field notes of sessions three, six, and nine (in the case of participant four, who had eight treatment sessions, we used sessions three, five, and seven). Based on the fidelity checklist, it was demonstrated that all the CO-OP principles listed in the checklist were addressed for each participant across sessions, indicating high treatment fidelity.

#### Technology Delivery

Two of the participants had no prior experience using a computer or tablet, and three of the participants were first time Skype™ users. In general, we were able to successfully communicate with the participants using the technology to carry out the intervention sessions as planned. Nevertheless, there were various technical problems we dealt with during the study. Skype™ and TalkHelper Call Recorder for Skype were not consistently reliable. The main issues were inadequate internet connection, and the difficulty some participants had of using the equipment and video conference software independently. Overall, of the total number of intervention sessions of all participants (53 sessions), there were video and/or audio disruptions in 14 sessions (26%), and only three sessions (6%) were either canceled or carried out via phone call due to severe technical issues. In order to solve the technical problems that arose during sessions, the OT provided technical support via telephone or was assisted by the participants' family members. Additionally, in two cases, the OT went to the participant's home and tried to solve the internet connection problem using Wi-fi amplifiers. Despite the technical issues described, all of the participants found the technological aspect of the intervention to be acceptable as described in the following acceptability section.

### Acceptability

Acceptability of the intervention was based on a satisfaction questionnaire and short feedback interviews post intervention. Overall, the participants were satisfied with the intervention. All five participants (100%) expressed high to very high satisfaction with the intervention in general and the therapeutic relationship. They all expressed their desire to continue the treatment if possible and stated that they would recommend this treatment to others with a similar health condition. Four out of five participants (80%) expressed high to very high satisfaction with the treatment process, which included the number, length, and frequency of sessions. Regarding the remote aspect of the intervention implementation, three participants (60%) expressed high to very high satisfaction with the remote nature of the treatment, and 80% were highly or very highly satisfied with the Skype™ software in terms of ease of use and quality of image and sound. Participant 2 expressed moderate satisfaction with the use of the software. It should be noted that despite the high satisfaction with the intervention, three of the five participants would have strongly preferred that the treatment be done face-to-face. Four participants (80%) expressed high to very high satisfaction with having a significant other involved in the treatment program and very high satisfaction with the level of involvement of their significant other. It should be noted, however, that one participant expressed very low satisfaction with having a significant other involved in the treatment program and medium satisfaction with the level of involvement of their significant other in the intervention process.

Some sample quotes from the feedback interviews illustrate the experience of participating in the intervention and give possible reasons for satisfaction with the intervention. When asked about the advantages of the intervention, one theme that was repeated was the functional improvements that followed the treatment. As participant 5 stated, “I [now] manage to put on clothes by myself… and it's true that it takes me 15 minutes to get dressed, but I don't get so frustrated.” Participants also reported an improvement in their sense of self-efficacy, as in this quote of participant 5, “[Now] I know that I can try it…[I know] that it's possible to get to where I need to go, to do what I need to do.” Participants and their significant others also positively described the process of guided discovery in the intervention. As the wife of participant 3 said, “She [the OT] told him, is it right or not? And what could he do to help himself?” Similarly, participant 5 explained, “If you ask and say, what is the difficulty? What can you do? What can we do to make it better?—this is very good. If at first, I don't succeed one way, I try another way, and maybe it will work.” When asked about the disadvantages of the intervention, participant 3 and the spouse of participant 5 mentioned that they would have liked to continue the intervention for a longer period of time. In addition, participant 1 reported that she felt that the required involvement of her significant other (her son) in the sessions was a burden to him.

When asked about their experience regarding the remote aspect of the intervention, Participant 3 stated, “[It] feels like face-to-face…in fact it is almost the same treatment [as face-to-face] because she saw me and I saw her.” Participant 5 said, “I think it would be nicer if it was face-to-face, but if you don't have the person face-to-face - it [the treatment] felt like face-to-face.” Participants mentioned that the use of the video conferencing provided an opportunity for the OT to see them performing activities in their natural environment. Participant 5 stated, “She watched it [what I did]…so I knew there was someone there who saw what I needed.” Participant 2 was the only one who reported during the feedback interview that there were technical issues which interfered with the treatment. Regarding the remote therapeutic relationship, participants expressed that they felt the OT gave them support that strengthened them and encouraged them to take action. Participant 1 said, “It gave me strength to do things that I could have postponed a lot more and maybe even given up on.” Participant 3 explained, “There is someone that is watching and looking out for you and trying to help with things that you find difficult and don't understand”.

### Preliminary Efficacy

#### Primary Outcome—Activity Performance in Participant-Chosen Goals

At baseline assessment, each participant identified five goals related to their daily function (see Table 2) and rated the importance of each goal they identified on a 10-point scale. Participants' chosen goals were in four main life domains according to the International Classification of Functioning, Disability and Health (ICF) activities and Participation component: (a) self-care, (b) domestic life, (c) major life areas, and (d) community, social, and civic life (99). Each Participant had goals in a few life domains with different variations. It should be noted that all participants chose at least one goal in the domain of community, social, and civic life.

**Table 2:** Participants' selected goals, importance ratings, and classification according to the ICF.

	**Goals**		**IR**	**Life domain-ICF**
P1	Trained	1. Exercise 1–2 times a week	10^*^	d5: Self-care- d5701: Managing diet and fitness
		2. Manage doctor appointments	10^*^	d5: Self-care- d5702: Maintaining one's health
		3. Finish embroidery artwork on blanket	10	d9: Community, social, and civic life- d920: Recreation and leisure
	Untrained	4. Manage bank account on-line	10^*^	d8: Major life areas- Economic life (d860-d870)
		5. Participate in a social leisure class	10^*^	d9: Community, social, and civic life- d920: Recreation and leisure, d9205: Socializing
P2	Trained	1. Return to community center	10	d9: Community, social, and civic life- d9205: Socializing, d920: Recreation and leisure
		2. Start cooking again	10^*^	d6: Domestic life- d630: Preparing meals
		3. Dress independently	10	d5: Self-care- d540: Dressing
	Untrained	4. Shower independently	10^*^	d5: Self-care- d510: Washing oneself
		5. Make a ponytail independently	10^*^	d5: Self-care- d5202: Caring for hair
P3	Trained	1. Start going to a community center	10^*^	d9: Community, social, and civic life- d9205: Socializing, d920: Recreation and leisure
		2. Dress lower body independently	10^*^	d5: Self-care- d540: Dressing
		3. Dry body after a shower independently	10^*^	d5: Self-care- d510: Washing oneself
	Untrained	4. Be more involved in managing health care	10	d5: Self-care- d5702: Maintaining one's health
		5. Independence in grocery shopping	10^*^	d6: Domestic life- d620: Acquisition of goods and services
P4	Trained	1. Do minor repairs at home	10^*^	d6: Domestic life- d6501: Maintaining dwelling and furnishings
		2. Find volunteer work	6^*^	d8: Major life areas- d855: Non-remunerative employment
		3. Go to lectures	8^*^	d9: Community, social, and civic life- d920: Recreation and leisure
		4. Go out with spouse for fun	10^*^	d9: Community, social, and civic life- d920: Recreation and leisure
		5. Visit children and grandchildren	10^*^	d9: Community, social, and civic life- d9205: Socializing
P5	Trained	1. Peel and cut vegetables	9^*^	d6: Domestic life- d630: Preparing meals
		2. Dress independently	10^*^	d5: Self-care- d540: Dressing
		3. Play with grandchildren on the floor	10^*^	d9: Community, social, and civic life- d9200: Play, d9205 Socializing
	Untrained	4. Go out with friend once a month	6^*^	d9: Community, social, and civic life- d920: Recreation and leisure, d9205: Socializing
		5. Exercise 1–2 times a week	5^*^	d5: Self-care- d5701: Managing diet and fitness

P, participant; IR, importance rating, rated by participants on a 10-point scale (1: not at all important, 10: very important) (74); ICF, the International Classification of Functioning, Disability and Health (http://apps.who.int/classifications/icfbrowser/).

* *Indicates goal improved to criterion (≥2 points) at post intervention and/or follow-up based on participants' COPM performance ratings (74)*.

During the intervention sessions, three of the five goals were trained directly using the CO-OP approach. The other two goals were not trained, thus enabling us to assess generalization and transfer of learning. One exception was participant 4, who worked directly on all five goals during the intervention sessions due to rapid progress with the first three goals. This participant was lost to follow-up due to an unstable medical condition unrelated to the study intervention. The goals are detailed in Table 2, and goals for which clinically meaningful improvements (≥2 points) were achieved based on participants' performance ratings on the COPM are indicated with an asterisk (*).

**Figure 2:**
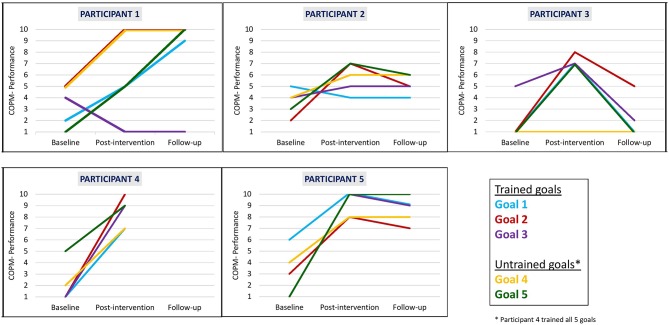
Participants' COPM performance scale ratings at baseline, post intervention, and 3-month follow-up.

**Figure 3:**
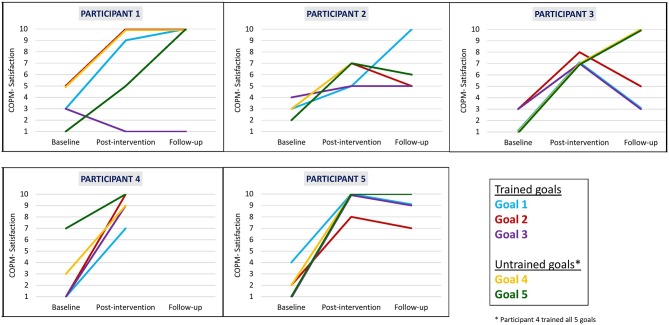
Participants' COPM satisfaction with performance scale ratings at baseline, post intervention, and 3-month follow-up.

We found that clinically meaningful improvements in performance and satisfaction ratings were achieved at post intervention and at follow-up in both trained and untrained goals. Overall, each participant improved in three to five of their five identified goals in both performance (see Figure 2) and satisfaction with performance (see Figure 3). Examination of the total clinically meaningful improvements indicates three main findings. First, the performance and satisfaction improvements that were achieved at post intervention were partially maintained at follow-up. The improvements of participants 1, 2, and 5 were maintained. However, few of participant 3's improvements were maintained. Second, satisfaction improvements were greater than performance improvements at both post intervention and at follow up and for both trained and untrained goals. Third, interestingly, a greater proportion of untrained goals than of trained goals showed improvements at both post intervention and at follow-up (see Table 3).

**Table 3:** The number of goals that reached a clinically significant improvement (≥2 points) on the COPM performance and satisfaction scales.

		**Trained goals**		**Untrained goals**		**All goals**	
		**Baseline-post**	**Baseline-FU**	**Baseline-post**	**Baseline-FU**	**Baseline-post**	**Baseline-FU**
Performance ratings	P1	2/3	2/3	2/2	2/2	4/5	4/5
	P2	1/3	1/3	2/2	2/2	3/5	3/5
	P3	3/3	1/3	1/2	0/2	4/5	1/5
	P4	5/5	–	–	–	5/5	–
	P5	3/3	3/3	2/2	2/2	5/5	5/5
	Total	14/17 (82.4%)	7/12 (58.3%)	7/8 (87.5%)	6/8 (75%)	21/25 (84%)	13/20 (65%)
Satisfaction ratings	P1	2/3	2/3	2/2	2/2	4/5	4/5
	P2	2/3	2/3	2/2	2/2	4/5	4/5
	P3	3/3	2/3	2/2	2/2	5/5	4/5
	P4	5/5	–	–	–	5/5	–
	P5	3/3	3/3	2/2	2/2	5/5	5/5
	Total	15/17 (88.2%)	9/12 (75%)	8/8 (100%)	8/8 (100%)	23/25 (92%)	17/20 (85%)

*COPM, the Canadian Occupational Performance Measure (74); Post, post intervention; FU, follow-up; P, participant*.

Wilcoxon Signed-Ranks test results (see Table 4) indicated statistically significant improvements from baseline to post intervention in COPM performance scale ratings on trained goals (*z* = −2.032, *p* = 0.042) and on all five goals (trained and untrained goals together, *z* = −2.023, *p* = 0.043). We found a near-significant improvement on the untrained goals (*z* = −1.841, *p* = 0.066). At follow-up, the COPM performance scores remained higher in comparison to baseline, and we found a near-significant improvement in trained goals and all goals (trained and untrained goals together, *z* = −1.826, *p* = 0.068 for both improvements). We found similar patterns for changes in satisfaction ratings at all time points. In addition, large effect sizes were demonstrated on changes in performance and satisfaction at post intervention and at follow-up.

**Table 4:** Changes in COPM median ratings from baseline to post intervention and from baseline to 3-month follow up (including effect size)^Θ^.

**Outcome measure (possible range of scores)**	**Baseline**	**Post-intervention**	**Baseline to** **post intervention** **(*****n*** **=** **5)**			**Follow-up**	**Baseline to** **follow-up** **(*****n*** **=** **4)**^**‡**^		
	**Mdn (IQR)**	**Mdn (IQR)**	***z***	***p***	***r* (ES)**	**Mdn (IQR)**	***z***	***p***	***r* (ES)**
**Performance (1–10)**	3.33 (2.17–3.67)	7.33 (5.33–8.87)	−2.032^*^	0.042	−0.909	5.67 (3.17–7.92)	−1.826^†^	0.068	−0.913
Trained goals (*n* = 5)									
Untrained goals (*n* = 4)^‡^	2.75 (1.38–3.38)	7.00 (4.63–8.63)	−1.841^†^	0.066	−0.921	7.50 (2.25–9.75)	−1.604	0.109	−0.802
All goals (*n* = 5)	3.00 (1.90–3.50)	6.20 (5.90–8.80)	−2.023^*^	0.043	−0.905	6.60 (2.80–8.45)	−1.826^†^	0.068	−0.913
**Satisfaction (1–10)**	2.60 (2.33–3.33)	7.33 (6.17–9.17)	−2.023^*^	0.043	−0.905	6.83 (4.42–8.00)	−1.826^†^	0.068	−0.913
Trained goals (*n* = 5)									
Untrained goals (*n* = 4)^‡^	2.00 (1.13–2.88)	7.25 (7.00–9.38)	−1.841^†^	0.066	−0.921	10.00 (7.00–10.00)	−1.826^†^	0.068	−0.913
All goals (*n* = 5)	2.60 (1.90–3.10)	7.20 (6.60–9.30)	−2.023^*^	0.043	−0.905	7.30 (6.25–8.80)	−1.826^†^	0.068	−0.913

COPM, the Canadian Occupational Performance Measure. Higher score reflects higher perceived performance/satisfaction with performance (74); Mdn, Median; IQR, interquartile range; EF, effect size.

Θ An effect size (r) was calculated from the z value of Wilcoxon signed-rank test ($r=z/\sqrt{}n$) (94) and can be interpreted as a small (r ≤ 0.10), medium (r = 0.30), and large (r ≥ 0.50) effect size (95).

* p < 0.05;

† near statistical significance; 0.05 ≤ p < 0.1.

‡ *Participant 4 was not included in this analysis (of untrained goals) because all his goals were trained during the intervention process; therefore, he had no untrained goals. In addition, this participant was lost to follow-up due to an unstable medical condition unrelated to the study intervention, and he was therefore not included in the baseline to follow-up analysis*.

#### Secondary Outcomes—Participation and Quality of Life

Participation was measured with the MPAI-4-P (79). Participation scores decreased (participation improved) from baseline (Mdn = 46.00, IQR = 42.25–59.50) to post intervention (Mdn = 33.50, IQR = 22.00–51.00). This improvement was near statistical significance (*z* = −1.826, *p* = 0.068). A similar trend was also observed when examining the changes in the participants' median MPAI-4-P scores at follow-up (Mdn = 36.00, IQR = 28.00–55.00), although the Wilcoxon Signed-Ranks test did not reveal a statistically significant difference from baseline (*z* = −1.604, *p* = 0.109). In addition, effect sizes were large at post intervention (*r* = −0.913) and at follow-up (*r* = −0.927).

**Figure 4:**
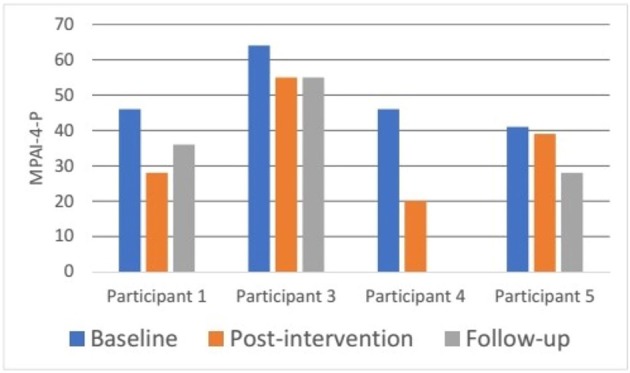
Participants' MPAI-4-P ratings at baseline, post intervention, and 3-month follow-up. MPAI-4-P, Mayo-Portland Adaptability Inventory-4, Participation Index (82).

Figure 4 shows participants' individual profiles of MPAI-4-P scores at baseline, post intervention, and follow-up. Participant 2 did not fill in the questionnaire due to technical issues and participant 4 was lost to follow-up due to an unstable medical condition unrelated to the study intervention. Participant 3 had the highest participation limitation at baseline (T score 64- reflecting severe participation limitation). The other three participants started at a similar level of participation limitation (T scores between 30 and 40, reflecting mild to moderate participation limitations). A decrease in MPAI-4-P scores was found for each participant from baseline to post intervention and from baseline to follow-up, suggesting greater participation and independence in community living. Furthermore, these differences reflect a clinical improvement in level of participation as described in the MPAI-4 manual (79).

Quality of life was measured with the SIS (82) at baseline, post intervention and follow up (see Table 5). Generally, no statistically significant improvement was found in the SIS subscales. However, near-statistically significant improvement was found on the “memory and thinking” subscale from baseline to post intervention and from baseline to follow-up (*z* = −1.841, *p* = 0.066 and *z* = −1.826, *p* = 0.068, respectively) with large effect sizes (*r* ≥ 0.80 for both). A near-significant improvement (*z* = −1.826, *p* = 0.068) was found on the “communication” subscale from baseline to post intervention, with a large effect size (*r* = −0.817); however, there was a decrease in follow-up scores, and these were not significantly different compared to baseline (*z* = −0.365, *p* = 0.715) and had a small effect size (*r* = −0.183).

**Table 5:** Changes in SIS median ratings from baseline to post intervention and from baseline to 3-month follow-up (including effect size)^Θ^.

**Outcome measure (possible range of scores)**	**Baseline**	**Post-intervention**	**Baseline to** **post intervention** **(*****n*** **=** **5)**			**Follow-up**	**Baseline to** **follow-up** **(*****n*** **=** **4)**^**‡**^		
	**Mdn (IQR)**	**Mdn (IQR)**	***z***	***p***	***r* (ES)**	**Mdn (IQR)**	***z***	***p***	***r* (ES)**
**SIS domains (0–100)**									
Strength	50.00 (28.50–75.00)	50.0 (34.50–78.50)	−0.0680	0.496	−0.030	47.00 (38.75–87.50)	−0.535	0.593	−0.268
Memory and thinking	68.00 (43.00–89.50)	89.00 (68.00–93.00)	−1.841^†^	0.066	−0.823	82.00 (52.25–98.25)	−1.826^†^	0.068	−0.913
Emotion	81.00 (54.50–94.00)	86.00 (71.00–90.00)	−0.535	0.593	−0.239	79.00 (63.75–94.25)	0.000	1.000	0.000
Communication	89.00 (73.50–96.50)	96.00 (89.00–98.00)	−1.826^†^	0.068	−0.817	80.00 (64.75–98.25)	−0.365	0.715	−0.183
ADL/IADL	80.00 (41.50–90.50)	75.0 (52.50–93.00)	−0.813	0.416	−0.364	70.0 (51.75–90.50)	−1.095	0.273	−0.548
Mobility	64.00 (44.50–86.00)	75.00 (69.50–86.00)	−1.289	0.197	−0.577	72.00 (61.75–90.50)	−0.736	0.461	−0.368
Hand function	50.00 (15.00–85.00)	70.00 (30.00–90.00)	−1.490	0.136	−0.666	55.00 (21.25–85.00)	−0.184	0.854	−0.092
Participation	69.00 (31.00–94.00)	84.00 (34.50–97.00)	−1.095	0.273	−0.490	56.00 (53.00–85.25)	−1.089	0.276	−0.545
General recovery	65.00 (45.00–85.00)	70.00 (45.00–82.50)	0.000	1.000	0.000	60.00 (27.50–92.50)	−0.365	0.715	−0.183

SIS, the stoke impact scale, higher scores reflect better QoL (82); QoL, quality of life; Mdn, Median; IQR, interquartile range; EF, effect size.

Θ An effect size (r) was calculated from the z value of Wilcoxon signed-rank test ($r=z/\sqrt{}n$) (94) and can be interpreted as a small (r ≤ 0.10), medium (r = 0.30), and large (r ≥ 0.50) effect size (95).

† near statistical significance; 0.05 ≤ p < 0.1.

‡ *Participant 4 was lost to follow-up due to an unstable medical condition unrelated to the study intervention, and he was therefore not included in the baseline to follow-up analysis*.

On an individual level, each participant achieved a clinically meaningful improvement of 15 points or more (88, 89) in one to seven (out of nine) subscales at post intervention and/or follow-up, suggesting improvement in different aspects of QoL. It should be noted that two participants reported clinically meaningful decreases in one or two subscales compared to baseline (along with improvements in other subscales).

## Discussion

This pilot study assessed the feasibility, acceptability, and preliminary efficacy of the CO-OP approach in a telerehabilitation format with adults in the chronic phase after ABI, prior to conducting a larger trial. Our findings indicated that implementation of the approach via videoconferencing is feasible and was found to be highly acceptable to the participants and their significant others. In addition, the study provided preliminary evidence of the intervention's efficacy. The most prominent improvements were found in the primary outcome of activity performance in the personal functional goals. Clinically meaningful improvements were also found in participation and QoL measures. Improvements were partially maintained at 3-month follow-up.

### Feasibility and Acceptability of the Intervention

We found that it was feasible to deliver the intervention remotely, while generally adhering to the essential elements of the CO-OP approach, and received reports of high satisfaction of participants. However, regarding the aspect of technology use for treatment delivery, there were difficulties similar to those reported in previous internet-based telerehabilitation studies (51, 52, 57, 100) and with tele-CO-OP specifically (24). Some difficulties might have been more prominent in the current study as the participants were relatively older adults with little experience in technology use and needed assistance to operate it (35, 101). Nevertheless, despite the technical challenges, all of the participants reported positive attitudes toward telerehabilitation, and this was in line with previous studies (102, 103). It is possible that these challenges affected recruitment rates and contributed to the relatively high dropout rates. In light of these issues, special consideration should be given to developing efficient ways of providing appropriate guidance and adequate technical support for various populations when planning telerehabilitation programs.

Four of the five final participants that completed the intervention showed good adherence rates, with appropriate numbers and duration of sessions. One exception is participant 4 who had several hospitalizations that led to breaks in the intervention process resulting in the extension of the intervention period to 8 months. Following the hospitalizations, the OTs debated whether to stop the intervention process. However, due to the participant's high motivation to take part in the home-based teleintervention and his recovery between hospitalizations, it was decided to continue the intervention. This case demonstrates the potential of the intervention to make a positive impact even in a case involving an unstable health condition, something that is prevalent in ABI survivors (104–106). This is particularly relevant for interventions with older adults who are considered a more vulnerable population (107).

Another factor that can affect the treatment process is the involvement of the significant other in the intervention program (108–110). In the current study, the significant others' involvement varied based on the participation needed in the participants' formulated plans as well as the willingness of the participants and the significant others to be involved. In some cases, the significant other mainly assisted in providing technical support, while in other cases they were involved in supporting the execution of the participants' plans. In addition, there was variation regarding the satisfaction of the participants with the involvement of the significant other in the treatment process. This highlights the complexity of this issue, as similarly discussed by Ng et al. (24). Despite the importance of the significant others' involvement in the rehabilitation process of adults with ABI and its potential positive effects, there are also barriers to this involvement, such as a lack of availability (108, 111, 112). When reflecting on the participants who dropped out during the study process, our impression was that they had less involvement or support from their significant others in regard to participating in the study.

### Preliminary Efficacy of the Intervention

Improving daily activity performance and participation is a valued and desired outcome in ABI rehabilitation, and there is a call to emphasize this aspect in outcome measures and as a focus of interventions (6, 10, 17). Accordingly, the primary outcome in our study was perceived performance and satisfaction on participant-chosen functional goals. Despite the small sample, significant statistical and clinical improvements were found in trained goals at post intervention and were partially maintained at follow up. These results are in line with previous studies that evaluated the efficacy of traditional face-to-face CO-OP approach among adults in the chronic stage post-ABI (113, 114). This improvement can be attributed to the client-centered, occupation-based nature of the intervention that focused directly on improving the performance of self-chosen goals. The intervention delivery to the participants in their natural environment, which is considered the ideal setting to address specific functional issues (20), may also have contributed to the gains in activity performance. Our results also indicated gains in the untrained goals suggesting the transfer of learning, similar to results in previous CO-OP studies (60, 61, 63, 73). These results can be explained by the metacognitive aspects of the approach and the emphasis on generalization and transfer during the sessions. This is assumed to facilitate participants' independent use of the global and domain-specific strategies in various life situations (115). In addition, participants' active involvement in the goal setting process is considered a motivational incentive that can promote goal attainment (116, 117). The improvements in activity performance and satisfaction were partially maintained at follow-up, as also reported in previous CO-OP studies that included adults with ABI (24, 60). Although activity participation and satisfaction improvements were maintained for participants 1, 2, and 5 at follow-up, participant 3 showed decreases in most of his goals at follow-up. These results might suggest that some participants require a longer intervention period or additional maintenance sessions to support achievements over time.

In relation to global outcomes of participation and QoL, the results were positive on the clinical level, yet less conclusive. This is similar to other studies that evaluated telerehabilitation interventions that target daily activities directly with ABI survivors, which have presented inconsistent or insufficient results regarding these outcomes (24, 52, 53, 55, 57). A possible explanation for this, in addition to the small sample size, is the rather short duration of the intervention, as well as the relatively low intensity of one weekly session (12, 117). Moreover, the current intervention focused on specific personal activities; consequently, the specific improvements in activity performance may not have been reflected significantly in the global participation and QoL measures, which include a broad range of life domains (52, 117).

### Limitations

The results need to be interpreted, taking into account the limitations of the study. First of all, these analyses were exploratory, and interpretation of the results was done with caution due to the small sample size and the possibility that the significant changes were found only by chance. Furthermore, the current study is a pilot study without a control group, which means we cannot attribute the improvements at post intervention solely to the treatment. However, it should be noted that none of the participants received additional occupational therapy during the intervention period. Therefore, we can assume that the activity performance improvements in the participant-chosen goals may be related to the studied intervention in general. Finally, we included a heterogenous group of participants with stroke and TBI with different levels of disability. While this limits the clear applicability to one group or the other, the fact that we found improvements in all patients suggests that this intervention has potential as a treatment for community dwelling individuals with chronic neurological conditions. In light of these limitations, further research is warranted with a larger sample and a control group. Additional outcome measures should be used to evaluate the effect of the intervention as perceived by the significant others and/or clinicians, as well as to deepen our understanding of who are the best candidates for this intervention.

## Conclusions

Improved functional performance is a main issue for many ABI survivors who continue to experience disability in a broad range of daily activities, and it is an important and desired outcome of rehabilitation. Our findings suggest that the delivery of the CO-OP approach via videoconferencing is feasible, acceptable, and beneficial to older adults in the chronic phase after TBI and stroke. Despite, the small and heterogeneous sample, we found significant improvements in activity performance as well as clinically meaningful improvements in activity performance, participation, and QoL for all of the participants. These improvements were partially maintained at 3-month follow up. Given accessibility barriers for receiving treatment in community-based clinics and the limited resources available for community in-home rehabilitation for ABI survivors, remotely delivered CO-OP could be a useful supplement to traditional rehabilitation options and could enable continued treatment for a longer period. Our encouraging results strengthen the evidence of the potential benefits occupation-based telerehabilitation interventions have in promoting activity performance, participation, and QoL among community dwelling ABI survivors in the long term. Based on these findings we are currently conducting a sufficiently powered randomized controlled study to further our understanding and strengthen the evidence of this intervention and its benefits.

## Data Availability Statement

The datasets analyzed in this manuscript are not publicly available. Requests to access the datasets should be directed to AB, aviva.kahan@mail.huji.ac.il.

## Ethics Statement

The studies involving human participants were reviewed and approved by the research ethics committees of Hadassah-Hebrew University Medical Center, Jerusalem and Maccabi Healthcare Services, Bat-Yam, Israel (ethical committee registration numbers: 0689-15-HMO and 192016, respectively). The patients/participants provided their written informed consent to participate in this study.

## Author Contributions

YG, JJ, AB, JS, YD, and IS conceived and designed the experiments. AB, SS, JS, FK, YN, and SB performed recruitment and data collection. AB, SS, and YG performed data analysis. AB wrote the manuscript. YG critically revised the manuscript. All the authors read and approved the final manuscript.

### Conflict of Interest

The authors declare that the research was conducted in the absence of any commercial or financial relationships that could be construed as a potential conflict of interest.
