# Supplementary material for: Activity Performance, Participation, and Quality of Life Among Adults in the Chronic Stage After Acquired Brain Injury—The Feasibility of an Occupation-Based Telerehabilitation Intervention
Source: Front Neurol. 2019 Dec 6;10:1247. doi: 10.3389/fneur.2019.01247 (PMC6908485; doi:10.3389/fneur.2019.01247)
Supplement: Supplementary file 1 [file Table_1.DOCX]

Supplementary Material

|  | 1  Very low | 2  Low | 3  Medium | 4  High | 5  Very high |
| --- | --- | --- | --- | --- | --- |
| 1. In general, how satisfied are you with the treatment program you have received? |  |  |  |  |  |
| 2. How much did you enjoy participating in the treatment program? |  |  |  |  |  |
| 3. How satisfied are you with the course of treatment (number and length of sessions, frequency of sessions)? |  |  |  |  |  |
| 4. How satisfied are you with the treatment approach used in the sessions (e.g. "Goal, Plan, Do, Check")? |  |  |  |  |  |
| 5. To what extent do you think you will continue to use the method you have learned to deal with other situations in your life? |  |  |  |  |  |
| 6. How satisfied are you with having a significant other (family member /formal caregiver / other) involved in the treatment program? |  |  |  |  |  |
| 7. How satisfied are you with the level of involvement of your significant other (family member /formal caregiver / other) in the treatment program? |  |  |  |  |  |
| 8. How satisfied are you with the therapeutic relationship between you and the occupational therapist during the treatment program? |  |  |  |  |  |
| 9. How satisfied are you with the remote treatment experience using video sessions? |  |  |  |  |  |
| 10. How satisfied are you with the experience of using Skype^TM^ in the treatment program (in terms of ease of use, quality of image and sound)? |  |  |  |  |  |
| 11. To what extent would you like to use this service again, if there were such a possibility? |  |  |  |  |  |
| 12. How likely are you to recommend our treatment program to a person with a similar health condition? |  |  |  |  |  |
| 13. To what extent would you prefer that the treatment had been done face-to-face? |  |  |  |  |  |

**Supplementary Figure 1.** Satisfaction with occupational therapy telerehabilitation intervention- Questionnaire items
